# Clinical and electrophysiological characteristics of myasthenia gravis patients with concomitant type 2 diabetes mellitus

**DOI:** 10.3389/fneur.2026.1776271

**Published:** 2026-02-20

**Authors:** Xue-Min Li, Yu-Dong Liu, Chun-Lin Yang, Peng Zhang, Yan-Mei Gong, Rui-Sheng Duan, Ying Liu

**Affiliations:** 1 Department of Neurology, Shandong Provincial Qianfoshan Hospital, Shandong University, Jinan, China; 2 Department of Neurology, The First Affiliated Hospital of Shandong First Medical University and Shandong Provincial Qianfoshan Hospital, Jinan, China; 3 Shandong Institute of Neuroimmunology, Jinan, China

**Keywords:** electrophysiology, myasthenia gravis, neuromuscular junction, repetitive nerve stimulation, type 2 diabetes mellitus

## Abstract

**Background and objectives:**

Type 2 Diabetes Mellitus (T2DM) is a common comorbidity in late-onset myasthenia gravis (MG), with pre-existing T2DM in some MG patients hinting at a possible pathogenic connection between the two disorders. This study aims to explore the clinical and neurophysiological features of MG, with a particular focus on the electrophysiological disparities between MG patients with and without T2DM.

**Methods:**

In this retrospective study, 170 MG inpatients were recruited and divided into T2DM (*n* = 51) and non-T2DM groups. Clinical profiles and electrophysiological characteristics were compared.

**Results:**

There were no significant differences between the two groups in terms of MG clinical classification, Quantitative MG (QMG) scores, or antibody profiles. Electrophysiologically, the T2DM group exhibited peripheral neuropathy, characterized by prolonged distal motor latencies, decreased compound muscle action potential (CMAP) amplitudes, slowed motor nerve conduction velocities, extended F-wave latencies, and sensory nerve dysfunction. In low-frequency repetitive nerve stimulation (LFRNS) of the facial nerve, the decrement pattern was predominantly “L-shaped” (minimal recovery) in T2DM patients, in contrast to the “U-shaped” pattern in non-T2DM patients. Accessory nerve LFRNS decrement showed a positive correlation with QMG score, fasting blood glucose levels, and diabetes duration.

**Conclusion:**

Patients with MG and concomitant T2DM demonstrate electrophysiological evidence of peripheral nerve impairment and a unique “L-shaped” decrement pattern on LFRNS of the facial nerve. This distinct electrophysiological phenotype likely reflects a complex interplay of metabolic derangements at the neuromuscular junction (NMJ). These results highlight the critical role of glycemic control in the management of MG.

## 1 Introduction

MG is an antibody-mediated autoimmune disorder that targets functional proteins on the postsynaptic membrane of the NMJ. Its primary pathophysiological mechanism is impaired neuromuscular transmission, manifesting clinically as fluctuating weakness and fatigability in skeletal muscles (1, 2). Epidemiological studies reveal a bimodal age distribution in MG onset. With an aging population, late-onset MG (LOMG, onset age >50 years) has become increasingly prevalent in clinical settings (3, 4).

Notably, T2DM is one of the most common comorbidities in patients with LOMG (5). Recent meta-analyses have demonstrated a significantly higher prevalence of T2DM among MG patients compared to the general population (6, 7). A case–control study further supports a strong association between T2DM and an increased risk of MG, suggesting that T2DM may serve as a potential risk factor for MG onset (8). This indicates a potential pathophysiological link between the two conditions that extends beyond mere coincidence. The underlying mechanisms of this association are multifaceted and not fully elucidated, potentially involving chronic hyperglycemia and insulin resistance-induced factors such as systemic low-grade inflammation, immune cell dysfunction, accumulation of advanced glycation end products, the effects of glucocorticoid therapy and reduced physical activity (9–14). A deeper understanding of the MG-T2DM comorbidity holds significant clinical value for optimizing patient management, improving prognosis, and identifying new therapeutic targets.

Neurophysiological studies, particularly Repetitive Nerve Stimulation (RNS) (15–18), are fundamental in the diagnosis and assessment of MG, providing objective evidence of “impaired neuromuscular transmission”. However, current clinical research has primarily focused on the electrophysiological characteristics of typical MG, with limited systematic investigation into potential electrophysiological alterations in MG patients with the important metabolic context of comorbid T2DM. Given that T2DM itself can cause Diabetic Peripheral Neuropathy (DPN) (19, 20) and may impact NMJ structure and function, the electrophysiological phenotype in the comorbid state is likely more complex, yet data in this area are scarce. Based on this research context, we designed and conducted a retrospective clinical study to explore the clinical and neurophysiological features of MG co-existing with T2DM.

## 2 Materials and methods

### 2.1 Patients and data sources

A retrospective study was conducted, encompassing all patients hospitalized for MG who underwent electromyographic evaluation at our center from February 2015 to January 2025. Individual-level data, such as demographics, clinical manifestations, comorbidities, auxiliary examination results and diagnoses, were extracted from electronic medical records.

### 2.2 Criteria of participants

MG diagnosis was confirmed based on international or Chinese diagnostic criteria (21), requiring typical fluctuating muscle weakness and at least one abnormal finding from pharmacological testing, electrophysiological examination or serum MG-related antibody detection, after excluding other possible diseases. Patients aged 50 years or older who experienced symptoms of muscle weakness for the first time were defined as LOMG. Thymoma-associated MG (TAMG) diagnosis required pathological results. Participants with T2DM were diagnosed in accordance with the Standards of Medical Care in Diabetes (Vision 2024) (22). Diagnostic information of MG or T2DM was cross-verified with discharge diagnosis or past medical history recorded in the EMR system.

It should be noted that patients with other conditions affecting the NMJ, muscles or peripheral nerves (excluding MG and diabetes) were excluded to prevent interference with electrophysiological results.

### 2.3 Neurophysiological evaluations

Neurophysiological evaluations were conducted by a certified biomedical technician using an electromyograph (Nihon Kohden). Surface electrodes were used for both stimulation and recording. Supramaximal stimulation was achieved by progressively increasing percutaneous electrical stimulus intensity to determine the level producing the maximal CMAP amplitude. Thereafter, the stimulus intensity was set at 20–30% above this level to ensure stable supramaximal stimulation throughout the recording. Motor nerve conduction studies assessed CMAP peak-to-peak amplitude, distal motor latency (DML), motor conduction velocity (MCV), and F-wave responses in the extremities. The median, ulnar, peroneal, and tibial nerves were stimulated, with CMAPs recorded from the abductor pollicis brevis, abductor digiti minimi, extensor digitorum brevis, and abductor hallucis muscles. Sensory nerve conduction studies measured sensory nerve action potential (SNAP) amplitudes and sensory conduction velocity (SCV) following stimulation of the median, ulnar, sural and peroneus superficialis nerves.

LFRNS of the facial nerve, ulnar nerve and accessory nerve were performed by delivering 10 electrical stimuli at 3 Hz, with surface recording electrodes placed at frontalis, orbicularis oculi, nasalis, mentalis, trapezius, and abductor digiti minimi. For low-frequency RNS (3 Hz), the decrement rate was calculated as [(Amplitude of the 4th CMAP–Amplitude of the 1st CMAP)/Amplitude of the 1st CMAP] × 100%. A decrement exceeding 10% in at least one muscle was deemed pathological. It is noteworthy that in RNS assessment, the amplitude of the CMAP was measured using the baseline-to-negative-peak amplitude. This adjustment was made due to the physiological characteristic of generally lower signal amplitudes in facial nerves. Given this, the continued use of the peak-to-peak measurement method could reduce accuracy and reliability due to the weaker signals. Additionally, patients taking acetylcholinesterase inhibitors were instructed to discontinue the medication 12–18 h prior to the examination, with their clinical condition taken into full consideration.

### 2.4 Quantitative analysis of LFRNS waveform morphology and dynamics

To objectively assess and compare LFRNS waveform morphology, we adapted an analysis method from prior research on NMJ disorders (23). A Recovery Index (RI) was calculated as CMAP₁₀ / CMAPₙₐ_dᵢ_ᵣ (CMAPₙₐ_dᵢ_ᵣ is the amplitude at the point of maximal decrement, typically the 4th or 5th response). RI < 1.02 indicated an “L-shaped” pattern (minimal recovery), while an index ≥ 1.02 indicated a “U-shaped” pattern (significant recovery). A piecewise linear mixed-effects model was employed to analyze the characteristics of amplitude ratio changes across different stimulation phases between the two groups. The 5th stimulus was defined as the turning point, dividing the stimulation sequence into two phases: stimuli 1–5 (Time 1) and stimuli 5–10 (Time 2). The amplitude ratio served as the dependent variable. The fixed effects included Time 1, Time 2, group (patients with T2DM vs. non-T2DM), and their interaction terms. Age as a covariate. Patient ID was included as a random effect in the model. Parameters were estimated using the restricted maximum likelihood method, and a *p* value < 0.05 was considered statistically significant.

### 2.5 Statistical analysis

All analyses were conducted using SPSS 27.0. Descriptive statistics summarized the distribution of variables in the study population. Categorical variables were compared using the *χ*^2^ test or Fisher’s exact test, while continuous variables were compared using the t-test or non-parametric tests. Repeated-measures analysis of variance was employed to evaluate the dynamic trend of the correlation data. Correlations between data sets were measured using Pearson and Spearman’s rank correlation coefficients. Given the significant age difference at onset, electrophysiological parameters were compared using ANCOVA, with age as a covariate. A *p*-value less than 0.05 was considered statistically significant.

### 2.6 Ethics approval

All nerve electrophysiological studies were performed by three experienced clinical neurophysiologists at our center, following a standardized protocol. Patients were fully informed of the necessity and potential discomfort associated with the tests before undergoing examinations, and written informed consent was obtained for all clinical data. This study was approved by the Research Ethics Committee of the First Affiliated Hospital of Shandong First Medical University.

## 3 Results

### 3.1 Cohort characteristics

A total of 170 MG patients were included in this study, with an average onset age of 56.17 years and 53.53% being female (Table 1). The time from initial myasthenic weakness onset to the current neurophysiological assessment exhibited considerable heterogeneity, reflecting both newly diagnosed patients and those with acute exacerbations. Symptom duration ranged from 3 days to 30 years, with a mean of 29.52 ± 54.90 months (Table 1). Based on clinical features, patients were categorized into subgroups: 71.18% had LOMG, 59.41% had GMG, and 88.82% had NTAMG (Table 1). Disease severity was evaluated using the QMG score. According to the Myasthenia Gravis Foundation of America (MGFA) classification, the majority of patients (86.47%) were classified as type I and II (Table 1). Approximately 95.15% of patients tested positive for MG-associated antibodies (Table 1).

**Table 1:** Demographic characteristics of MG patients.

Characteristics (*n*)	Cases *n* = 170 (%)
Male: Females (170)	79: 91
Age of onset (years: mean ± sd)	56.17 ± 17.73
Time from MG onset to testing (months: mean ± sd)	29.52 ± 54.90
EOMG/LOMG (170)	
EOMG	49 (28.82)
LOMG	121 (71.18)
OMG/GMG (170)	
OMG	69 (40.59)
GMG	101 (59.41)
TAMG/NTAMG (170)	
TAMG	19 (11.18)
NTAMG	151 (88.82)
MGFA clinical type (170)	
I	69 (40.59)
II	78 (45.88)
III	16 (9.41)
IV	3 (1.76)
V	4 (2.35)
MG antibodies (103)	
Positive	98 (95.15)
Negative	5 (4.85)

QMG, Quantitative Myasthenia Gravis; EOMG, Early-Onset Myasthenia Gravis; LOMG, Late-Onset Myasthenia Gravis; OMG, Ocular Myasthenia Gravis; GMG, Generalized Myasthenia Gravis; TAMG, Thymoma-Associated Myasthenia Gravis; NTAMG, Non-Thymoma-Associated Myasthenia Gravis; MGFA, Myasthenia Gravis Foundation of America.

### 3.2 Clinical and electrophysiological characteristics of non-diabetic and diabetic MG patients

In this study, 51 MG patients (30.00%) suffered from T2DM, with an average duration of 7.96 ± 8.68 years. The clinical and electrophysiological features of diabetic and non-diabetic MG patients were subsequently analyzed.

#### 3.2.1 Clinical characteristics

The gender distribution showed no significant difference between the two groups, though the proportion of males was slightly higher in the diabetic group. The mean age at disease onset was significantly higher in diabetic MG patients compared with non-diabetic patients (63.65 ± 8.94 vs. 52.97 ± 19.53 years, *p* < 0.01; Table 2). The proportion of LOMG was also significantly higher in the diabetic group than in the non-diabetic group (94.12% vs. 61.34%, *p* < 0.001; Table 2). No significant difference was found in the classification of OMG/GMG or NTAMG/TAMG. Similarly, MGFA classification did not differ significantly. The proportion of patients with thymic abnormalities, particularly thymic hyperplasia, was significantly lower in the diabetic group than in the non-diabetic group (*p* < 0.05; Table 2). No differences were found in the types of MG-related antibodies between the two groups. Disease severity, as assessed by the quantitative myasthenia gravis (QMG) score, showed no significant difference.

**Table 2:** Different characteristics between non-diabetic and diabetic MG patients.

Clinical characteristics	T2DM −	T2DM +	Available number	*P*
*n*	119	51	170	
Gender, Males (%)	52 (43.70)	27 (52.94)	170	ns
Onset age (mean ± sd)	52.97 ± 19.53	63.65 ± 8.94	170	< 0.01
LOMG (%)	73 (61.34)	48 (94.12)	170	< 0.001
OMG (%)	48 (40.34)	21 (41.18)	170	ns
TAMG (%)	16 (13.01)	3 (6.00)	170	ns
MGFA clinical type (%)				
I	48 (40.34)	21 (44.18)	170	ns
II	52 (43.70)	26 (50.98)		
III	13 (10.92)	3 (5.88)		
IV	3 (2.52)	0 (0)		
V	3 (2.52)	1 (1.96)		
Thymus				
Normal	75 (63.03)	42 (82.35)	170	< 0.05
Thymic hyperplasia	28 (23.53)	6 (11.76)		
Thymoma	16 (13.45)	3 (5.88)		
Positive MG antibodies (%)	66 (95.65)	32 (94.12)	98	ns
Anti-AchR (+)	59 (85.51)	32 (94.12)	98	ns
Anti-RYR (+)	6 (8.70)	2 (5.88)	98	ns
Anti-MuSK (+)	4 (5.80)	0 (0)	98	ns
Anti-Tltin (+)	25 (36.23)	15 (44.12)	98	ns
Anti-LRP4 (+)	2 (2.90)	0 (0)	98	ns
QMG scores	10.26 ± 4.67	10.48 ± 5.07	91	ns

QMG, Quantitative Myasthenia Gravis; LOMG, Late-Onset Myasthenia Gravis; OMG, Ocular Myasthenia Gravis; TAMG, Thymoma-Associated Myasthenia Gravis; MGFA, Myasthenia Gravis Foundation of America; AChR-Ab, Acetylcholine Receptor Antibody; MuSK-Ab, Muscle-Specific Kinase Antibody; LRP4-Ab, Low-Density Lipoprotein Receptor-Related Protein 4 Antibody; Titin-Ab, Titin Antibody; RyR-Ab, Ryanodine Receptor Antibody.

#### 3.2.2 Electrophysiological characteristics

In this study, a total of 164 patients underwent electrophysiological examinations.

##### 3.2.2.1 Nerve conduction studies

The T2DM patients were older than those in the non-diabetic group. Given that age independently affects electrophysiological parameters, such as the well-established decline in CMAP amplitude with age across multiple nerves (24, 25), age was included as a covariate in ANCOVA for all intergroup comparisons.

Motor nerve conduction studies (Table 3) showed that the initially significant unadjusted differences in median nerve CMAP amplitude (*p* = 0.023) and median nerve MCV (*p* = 0.042) lost statistical significance after age adjustment (*p* > 0.05). However, the T2DM group still showed a persistent and statistically significant prolongation of median nerve DML (adjusted *p* = 0.022), reduction in tibial nerve CMAP amplitude (adjusted *p* = 0.032), and slowing of peroneal nerve MCV (adjusted *p* = 0.015). Regarding F-wave latencies, the prolongations in the median and ulnar nerves became non-significant after age adjustment, but a significant prolongation remained in the tibial nerve (adjusted *p* = 0.02).

**Table 3:** Comparison of nerve conduction parameters between non-T2DM and T2DM groups before and after age adjustment.

Electrophysiological parameters	Non-T2DM (Mean ±SD)	T2DM (Mean ±SD)	Unadjusted *p*	Age-adjusted *p*	Remarks
Motor nerve conduction					
Median nerve CMAP (mV)	11.38 ± 3.36	10.19 ± 2,35	**0.023**	0.315	NS
Ulnar nerve CMAP (mV)	13.11 ± 3.05	12.57 ± 3.41	0.371	0.410	NS
Peroneal nerve CMAP (mV)	7.07 ± 3.50	5.62 ± 1.62	0.091	0.386	NS
Tibial nerve CMAP (mV)	18.19 ± 7.65	14.28 ± 6.51	**0.01**	**0.032**	Significant
Median nerve DML (ms)	3.24 ± 2.71	3.46 ± 0.72	**0.003**	**0.022**	Significant
Ulnar nerve DML (ms)	2.40 ± 0.36	2.49 ± 0.36	0.215	0.803	NS
Peroneal nerve DML (ms)	3.73 ± 0.75	3.83 ± 0.75	0.516	0.917	NS
Tibial nerve DML (ms)	3.50 ± 0.50	3.61 ± 0.67	0.965	0.862	NS
Median nerve MCV (m/s)	57.18 ± 4.79	55.39 ± 4.04	**0.042**	0.537	NS
Ulnar nerve MCV (m/s)	59.50 ± 5.42	56.53 ± 6.01	0.057	0.298	NS
Peroneal nerve MCV (m/s)	48.96 ± 4.49	44.74 ± 4.96	**0.001**	**0.015**	Significant
F-wave					
Median nerve F-wave (ms)	24.85 ± 2.34	25.72 ± 2.16	**0.05**	0.249	NS
Ulnar nerve F-wave (ms)	25.23 ± 2.41	26.15 ± 2.10	**0.042**	0.168	NS
Tibial nerve F-wave (ms)	45.77 ± 3.64	49.01 ± 5.01	**0.002**	**0.02**	Significant
Sensory nerve conduction					
Median nerve (thumb) SNAP (μV)	21.49 ± 0.83	15.44 ± 7.44	**0.006**	**0.014**	Significant
Median nerve (middle finger) SNAP (μV)	17.92 ± 7.93	12.43 ± 5.97	**<0.001**	**0.016**	Significant
Ulnar nerve SNAP (μV)	12.64 ± 6.83	9.98 ± 2.48	**0.001**	0.173	NS
Superficial peroneal nerve SNAP (μV)	11.49 ± 6.07	8.44 ± 5.23	0.115	0.464	NS
Sural nerve SNAP (μV)	13.22 ± 7.54	9.48 ± 4,54	0.053	0.264	NS
Median nerve (thumb) SCV (m/s)	51.18 ± 7.15	46.23 ± 8.90	**0.003**	0.077	NS
Median nerve (middle finger) SCV (m/s)	54.77 ± 6.87	49.90 ± 8.69	**<0.001**	**0.021**	Significant
Ulnar nerve SCV (m/s)	56.14 ± 5.57	54.71 ± 8.23	0.255	0.735	NS
Superficial peroneal nerve SCV (m/s)	52.14 ± 6.69	49.56 ± 5.30	0.059	0.281	NS
Sural nerve SCV (m/s)	52.40 ± 6.68	49.30 ± 5.81	**0.032**	0.131	NS

DML, Distal Motor Latency; MCV, Motor Conduction Velocity; SNAP, Sensory Nerve Action Potential; SCV, Sensory Conduction Velocity; CMAP, compound muscle action potential. Bold values indicate statistical significance (*p* < 0.05).

Sensory nerve conduction studies revealed more consistent findings. After age adjustment, the type 2 diabetes group maintained significantly lower median SNAP amplitudes (thumb: *p* = 0.014; middle finger: *p* = 0.016) and slower median SCV at the middle finger (*p* = 0.021). Conversely, sensory parameters for the ulnar and superficial peroneal nerves indicated no significant intergroup differences after age correction (Table 3).

In summary, age-adjusted analysis provided more precise results, indicating that median nerve abnormalities and specific motor deficits in the tibial and peroneal nerves are stable electrophysiological features of T2DM independent of age. Although prolonged median DML and sensory abnormalities suggest carpal tunnel syndrome, the coexisting length-dependent sensorimotor polyneuropathy in the lower limbs and prolonged F-wave latencies in the legs indicate underlying DPN.

##### 3.2.2.2 Repetitive nerve stimulation

**Figure 1:**
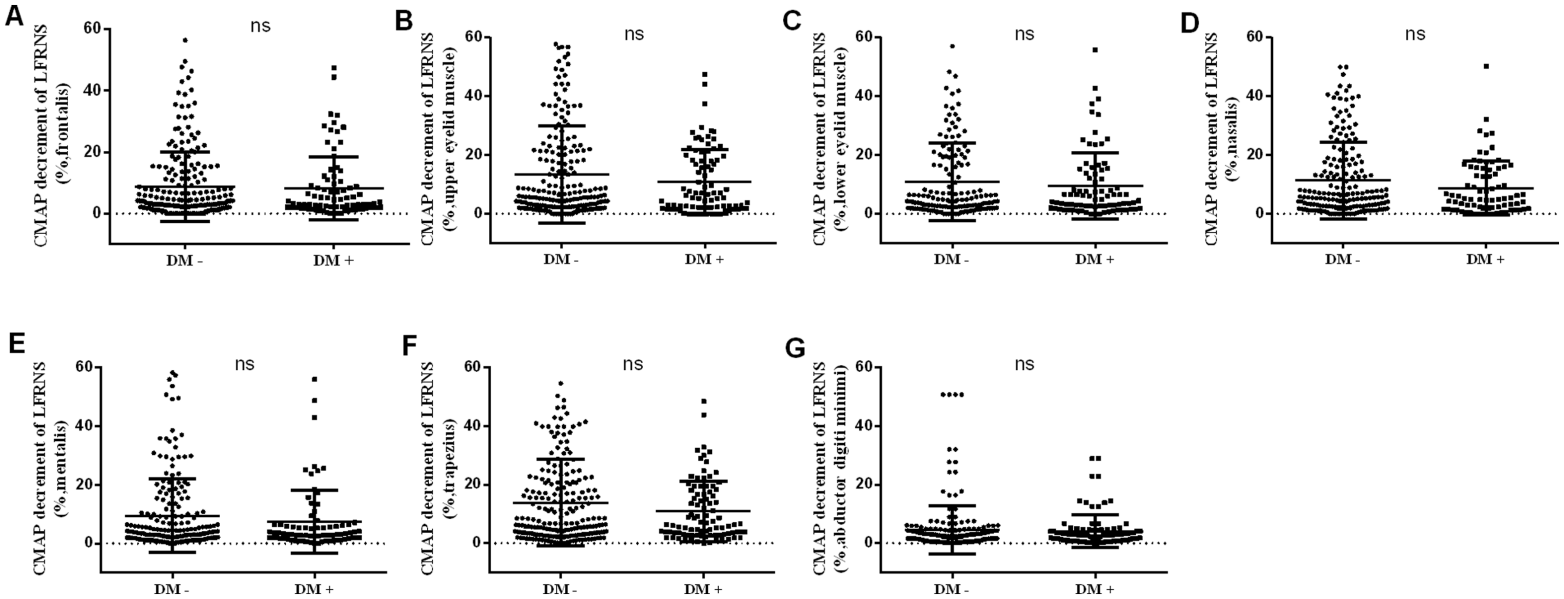
The characteristics of LFRNS between non-diabetic and diabetic MG patients. There was no statistical difference in the CMAP decrement of LFRNS between non-diabetic and diabetic MG patients **(A–G)**. (CMAP, compound muscle action potential; LFRNS, low-frequency repetitive nerve stimulation).

The positivity rate of LFRNS was 70.69% (82/116) in the non-diabetic group and 70.83% (34/48) in the diabetic group, with no significant difference. Similarly, no significant difference was observed in the degree of low-frequency decrement across different nerves between the two groups (Figures 1A–G).

**Figure 2:**
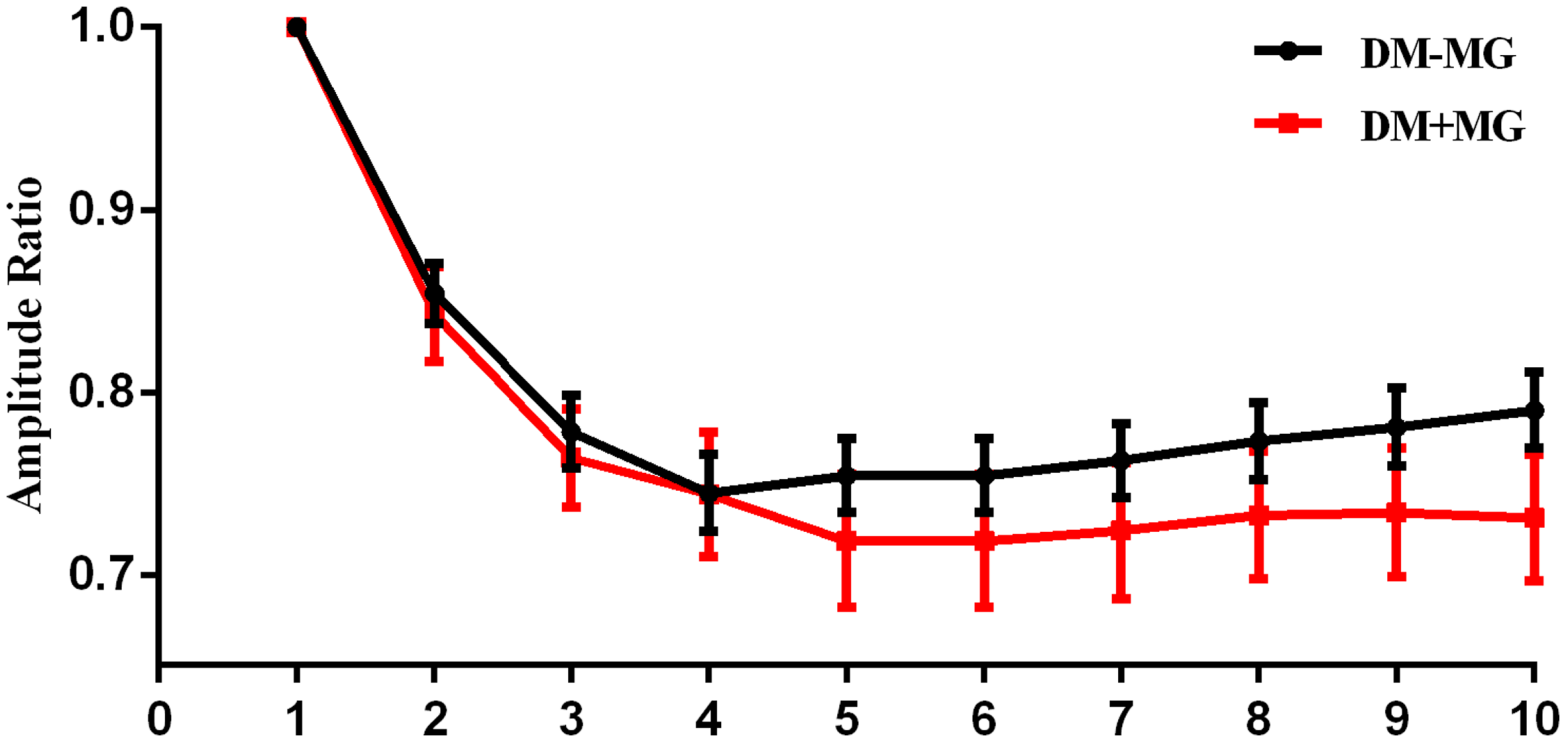
Amplitude ratio curve of non-diabetic and diabetic MG’s facial nerve. For non-diabetic MG patients’ facial nerve, the lowest CMAP appeared in the 4th at 3 Hz RNS and recovered significantly since the 7th CMAP; for diabetic MG patients’ facial nerve, the lowest CMAP appeared in the 5th at 3 Hz RNS and there was no significant recovery after the nadir of decrement (RNS, repetitive nerve stimulation).

This study compared the electrophysiological characteristics of non-T2DM and T2DM MG patients by analyzing the amplitude ratio of the 1st to the 10th CMAP during LFRNS of the facial and accessory nerves. A key finding was the distinct patterns of CMAP recovery observed in the facial nerve (mentalis muscle recording) between the two groups (Figure 2).

Detailed electrophysiological data revealed that non-diabetic MG patients exhibited a classic “U-shaped” recovery pattern. The CMAP decrement reached its nadir at the 4th stimulus, followed by significant recovery, yielding a recovery index (RI, calculated as CMAP_10_/CMAP_nadir_) of 1.06 ± 0.04. In contrast, diabetic MG patients displayed an “L-shaped” pattern. The maximal decrement occurred at the 5th stimulus, with no subsequent significant recovery (RI = 1.01 ± 0.02). The difference in RI between the two groups was statistically significant (*p* < 0.001, Table 4). Categorical analysis of individual patient patterns further demonstrated that 83.3% of non-diabetic patients conformed to the U-shaped pattern, while 62.5% of diabetic patients exhibited the L-shaped pattern, a distribution that was significantly different (*p* < 0.001). In terms of statistical analysis, while the generalized estimating equations (GEE) model did not show an overall between-group difference (*p* > 0.05), it revealed a significant main effect of stimulus number (Wald *χ*^2^ = 293.299, *p* < 0.001) and a significant group-by-stimulus interaction effect (Wald *χ*^2^ = 80.640, *p* < 0.001). Post-hoc comparisons with Bonferroni correction (Table 4) indicated that the non-diabetic group showed significant recovery at stimuli 7 through 10 compared to the nadir (*p* < 0.0056), whereas no such change was observed in the diabetic group. To precisely quantify the difference in recovery trends following the 5th stimulus, a piecewise linear mixed-effects model incorporating subject-level random effects was constructed. The results demonstrated a non-significant group-by-Time1 (pre-5th stimulus) interaction (*F* = 0.105, *p* = 0.746), but a significant group-by-Time2 (post-5th stimulus) interaction (*F* = 5.686, *p* = 0.018) (Table 5). This confirms that the recovery slope after the maximal decrement was significantly lower in diabetic MG patients for the facial nerve. These findings suggest that minimal recovery of CMAP amplitude following the nadir of decrement in the facial nerve of diabetic MG patients.

**Table 4:** Comparison of LFRNS parameters between non-T2DM and T2DM groups.

Parameters	Non-T2DM (Mean ± SD)	T2DM (Mean ± SD)	*p*
Number of stimulations			
1st	1.00 ± 0.00^*^	1.00 ± 0.00^*^	0.935
2st	0.85 ± 0.09^*^	0.85 ± 0.11^*^	0.791
3st	0.78 ± 0.11^*^	0.79 ± 0.11^*^	0.457
4st	0.75 ± 0.11	0.77 ± 0.13^*^	0.973
5st	0.75 ± 0.11	0.75 ± 0.13	0.973
6st	0.75 ± 0.11	0.75 ± 0.13	0.973
7st	0.76 ± 0.12^*^	0.76 ± 0.13	0.833
8st	0.77 ± 0.11^*^	0.76 ± 0.13	0.731
9st	0.78 ± 0.12^*^	0.76 ± 0.12	0.582
10st	0.79 ± 0.11^*^	0.76 ± 0.12	0.935
$\frac{\text{CMAP}10}{\text{CMAPnadir}}$	1.06 ± 0.04	1.01 ± 0.02	<0.001^☨^
L Shape (%)	5 (17.6)	25 (83.3)	**0.003** ^**#**^
U Shape (%)	10 (62.5)	6 (37.5)	

CMAP, compound muscle action potential; LFRNS, Low-frequency repetitive nerve stimulation.

Values are presented as mean ± standard deviation (or other appropriate descriptive statistics).

☨ *p* < 0.001, significant difference between groups.

# *p* = 0.003, significant difference in the distribution of L-shaped vs. U-shaped patterns between groups (chi-square test).

^*^p < 0.0056, significant difference compared to the nadir point within the same group. Bold values indicate statistical significance (*p* < 0.05).

**Table 5:** Parameter estimates from the piecewise linear mixed-effects mode.

Parameters	Estimate (*b*)	SE	95% CI	*F*	*p*
Intercept	0.944	0.026	[0.890, 0.990]	3315.390	<0.001
Group	−0.004	0.033	[−0.070, 0.060]	0.017	0.896
Time 1	−0.053	0.004	[−0.060, −0.460]	602.041	<0.001
Time 2	0.008	0.003	[0.002, 0.013]	45.357	<0.001
Group × Time 1	−0.001	0.004	[−0.01, 0.007]	0.105	0.746
Group × Time 2	0.008	0.003	[0.001, 0.015]	5.686	**0.018**

Time 1, Trials 1–5; Time 2, Trials 5–10; Group, non-T2DM and T2DM Groups; Reference group, non-T2DM. Bold values indicate statistical significance (*p* < 0.05).

### 3.3 Correlation between electrophysiological results and clinical features

**Figure 3:**
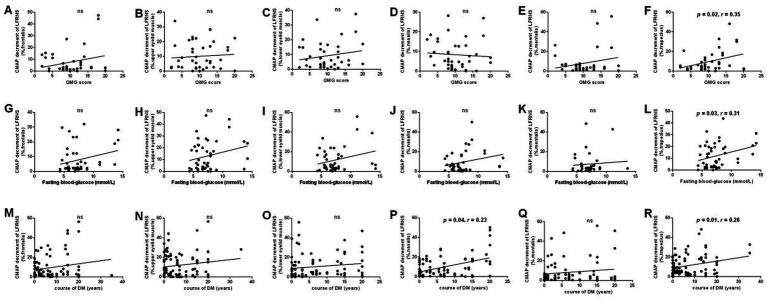
Correlation analysis between CMAP decrement on LFRNS and clinical parameters. Correlation analysis between CMAP decrement on LFRNS and QMG scores **(A-F)**, fasting blood-glucose **(G-L)**, and DM durations **(M-R)**. CMAP decrement of LFRNS of accessory nerve significantly positively correlated with QMG scores **(F)**. CMAP decrement of LFRNS of accessory nerve significantly positively correlated with fasting blood-glucose **(L)**. CMAP decrement of LFRNS of accessory nerve and facial nerve significantly positively correlated with DM duration **(P, R)**. (CMAP, compound muscle action potential; LFRNS, low-frequency repetitive nerve stimulation, quantitative myasthenia gravis) (^*^p < 0.05).

Our results indicated a positive correlation between QMG score and the degree of low-frequency decrement in the accessory nerve (Figure 3F), indicating that CMAP decrement of LFRNS significantly correlates with clinical symptom severity. Fasting blood glucose, a key diagnostic indicator for diabetes, measured after 8–12 h of fasting, was positively correlated with LFRNS CMAP decrement in the accessory nerve in the diabetic cohort (Figure 3L), with higher glucose levels associated with greater reductions. Additionally, among diabetic patients, the duration of DM was positively correlated with the degree of CMAP decrement on LFRNS in both the accessory and facial nerves (Figures 3P,R).

## 4 Discussion

This retrospective study compared the clinical and electrophysiological characteristics of MG patients with and without comorbid T2DM. The key finding is that MG patients with T2DM displayed a characteristic L-shaped decrement pattern during LFRNS at a specific nerve-muscle site (facial nerve–mentalis muscle). This decrement correlated with fasting blood glucose levels, diabetes duration, and clinical symptom severity. Additionally, MG patients with T2DM were more prone to peripheral nerve conduction abnormalities.

Diabetes has been proposed as a potential risk factor for the onset of MG (8), though the underlying mechanisms remain incompletely understood. Chronic hyperglycemia may induce immune dysregulation, including impaired Ca^2+^signaling and aberrant immune responses under high-glucose conditions (26–28). Advanced glycation end-products can activate the receptor for AGEs pathway, leading to the release of pro-inflammatory cytokines and proliferation of autoimmune-related cell subsets like CD4(+)CD28(−) T cells, contributing to MG pathogenesis (29, 30). Our group’s prior animal studies further showed that a T2DM environment can drive the differentiation and activation of follicular helper T cells, exacerbating humoral immune abnormalities and creating a predisposition for MG development (12, 13). Thus, routine screening for glucometabolic status is recommended for patients with LOMG.

In the present study, the observed positive rate of Antibody positivity in MG exceeded the commonly reported ranges in previous literature. The notably high overall antibody positivity rate observed in our center may be attributed to several factors, including the adoption of highly sensitive detection methods (cellular immunofluorescence assay and cell-based assay) and the routine screening for a broad array of antibodies, including Acetylcholine Receptor Antibody (AChR-Ab), Muscle-Specific Kinase Antibody (MuSK-Ab), Low-Density Lipoprotein Receptor-Related Protein 4 Antibody (LRP4-Ab), Titin Antibody (Titin-Ab) and Ryanodine Receptor Antibody (RyR-Ab). Additionally, potential differences in patient enrollment criteria may also have influenced these findings. To explore the morphological characteristics of LFRNS, this study focused on the facial nerve (recorded at the mentalis muscle) and the accessory nerve (recorded at the trapezius muscle) as the observation targets. This selection was guided by methodological considerations: traditional facial nerve recordings are often hindered by technical variability due to the low amplitude and instability of responses in commonly targeted muscles under repetitive stimulation. In contrast, the mentalis muscle (innervated by the mandibular branch of the facial nerve) and the trapezius muscle (innervated by the accessory nerve) produce more stable and reproducible CMAP waveforms during repetitive electrical stimulation. By using these nerves as experimental models, we aimed to reduce technical variability, improve the reliability of RNS morphological data, and strengthen the validity and persuasiveness of our conclusions.

The most salient electrophysiological finding in this study is the morphological difference in decrement curves. Classic MG typically presents with a negative decrement, where the CMAP amplitude shows significant recovery at the 6th or 7th stimulus during RNS, forming a “U-shaped curve” (31) In the present study, however, we observed an “L-shaped” LFRNS curve in facial nerve-mentalis recordings from MG patients with comorbid T2DM-characterized by an initial amplitude decline after stimulation, followed by a plateau without the typical U-shaped recovery. This pattern is rare in classic postsynaptic MG but resembles disorders with presynaptic or pan-synaptic involvement, such as Lambert-Eaton myasthenic syndrome (LEMS) and amyotrophic lateral sclerosis (ALS) (23, 32–34). It is suggested that the distinct trends of U-shaped and L-shaped curves reflect different underlying mechanisms of NMJ dysfunction. In ALS, the impairment may involve the entire NMJ structure, including both presynaptic and postsynaptic components (35, 36), while in LEMS, it primarily relates to a defect in acetylcholine release, where the physiological reduction in release induced by RNS cannot be compensated by reserve pools (34, 37). This suggests that the mechanism of neuromuscular transmission impairment in MG with T2DM may be more complex.

A plausible explanation is the combined effect of DPN and DM-related injury on the NMJ. MG patients with T2DM in this study exhibited peripheral nerve conduction abnormalities, including prolonged DML, reduced CMAP amplitude, slowed conduction velocity, and prolonged F-wave latency in the lower limbs, consistent with the electrophysiological profile of DPN. In diabetic patients, axonal damage may lead to impaired axoplasmic transport, which can result in disrupted transport of acetylcholine from its site of synthesis in the cell body to the axonal terminals, ultimately causing decreased acetylcholine storage in synaptic vesicles. Hyperglycemia and its downstream effects, such as oxidative stress, mitochondrial dysfunction, and microangiopathy, collectively contribute to axonal and myelin damage in peripheral nerves (38–40). At the NMJ level, both animal and clinical studies indicate that diabetes can alter axonal morphology, NMJ ultrastructure, and motor function (41, 42). Specific ultrastructural abnormalities, including T-tubule disruption, decreased synaptic vesicle number, and mitochondrial swelling with irregular cristae, have been associated with neuromuscular transmission deficits (43–45). Such presynaptic and/or axonal-level damage, along with NMJ ultrastructural alterations, may interact with or superimpose upon the inherent postsynaptic acetylcholine receptor deficiency in MG, collectively contributing to the impaired recovery characteristic of the “L-shaped” decrement pattern. However, this study did not employ systematic DPN clinical grading or advanced assessments, making comparative RNS analysis stratified by DPN severity unfeasible. This limitation warrants investigation in future stratified studies.

It should be emphasized that this distinct “L-shaped” pattern was primarily observed in the facial nerve-mentalis muscle configuration in the present study. The relatively low amplitude in this region may introduce technical artifacts, so this finding should be regarded as an electrophysiological phenotype emerging under specific conditions, namely, in cases of MG coexisting with T2DM and limited to a particular detection site. It does not represent a general characteristic of MG or diabetic neuromuscular pathology and should not be overgeneralized.

Correlation analysis revealed that the degree of low-frequency RNS decrement in the facial nerve was positively correlated not only with the QMG score, reflecting MG severity, but also with fasting blood glucose levels and the duration of diabetes. This further supports the hypothesis that a hyperglycemic environment may exacerbate neuromuscular transmission dysfunction. However, it must be clearly recognized that, due to the cross-sectional design of this study, these correlations do not establish causality. Poor glycemic control and MG disease activity may interact and serve as confounding factors. For instance, severe MG may reduce activity and corticosteroid use, thereby affecting glycemic control; conversely, chronic hyperglycemia may worsen neuromuscular symptoms through the mechanisms described above. This association highlights the clinical importance of blood glucose monitoring and management in MG patients, though exact causal pathways require clarification through prospective studies.

This study has several limitations. First, the retrospective single-center design may introduce selection bias. Second, the limited sample size, particularly in the T2DM subgroup, may affect the power of certain subgroup analyses. Third, as noted earlier, the lack of systematic assessment and stratification of DPN complicates disentangling its influence on electrophysiological outcomes. Fourth, although standardized procedures were followed, RNS recordings from the facial nerve-mentalis muscle are technically challenging and may introduce variability. Fifth, the cross-sectional nature of the data precludes inference of temporal or causal relationships between glycemic parameters and MG characteristics. Finally, the absence of healthy controls or a T2DM-only (without MG) control group limits our understanding of the specificity of the observed phenomena. Future studies could employ more sensitive techniques, including single-fiber electromyography combined with prospective designs, larger samples, and systematic neuropathy evaluation, to validate and extend these findings.

## 5 Conclusion

In summary, from an electrophysiological perspective, MG patients with comorbid T2DM not only exhibit peripheral nerve conduction abnormalities but may also demonstrate a distinctive “L-shaped” LFRNS decrement pattern at the facial nerve. This pattern likely reflects the complex interplay at the NMJ between hyperglycemia-related metabolic neuropathy and autoimmune postsynaptic membrane disease. The observed correlations between blood glucose levels, diabetes duration, and the degree of RNS decrement underscore the importance of monitoring glycemic metabolism in managing MG patients. Our findings provide new insights into how metabolic factors may modulate the electrophysiological phenotype of autoimmune neuromuscular disorders, though the underlying mechanisms and clinical implications require further investigation.

## Data Availability

The raw data supporting the conclusions of this article will be made available by the authors, without undue reservation.

## References

[1] Tannemaat MR Huijbers MG Verschuuren J. Myasthenia gravis-pathophysiology, diagnosis, and treatment. Handb Clin Neurol. (2024) 200:283–305. doi: 10.1016/B978-0-12-823912-4.00026-8, 38494283

[2] Pasnoor M Wolfe GI Barohn RJ. Myasthenia gravis. Handb Clin Neurol. (2024) 203:185–203. doi: 10.1016/B978-0-323-90820-7.00006-9, 39174248

[3] Hoffmann S. Special populations in myasthenia gravis: early, late, and very late-onset Mg. Int Rev Neurobiol. (2025) 182:197–204. doi: 10.1016/bs.irn.2025.04.032, 40675735

[4] Mishra AK Varma A. Myasthenia gravis: a systematic review. Cureus. (2023) 15:e50017. doi: 10.7759/cureus.50017, 38186498 PMC10767470

[5] Croitoru CG Pavel-Tanasa M Cuciureanu DI Hodorog DN Cianga P. Autoimmune and non-autoimmune comorbidities in myasthenic patients of east-European descent: a case-control study. J Clin Med. (2024) 13:2273. doi: 10.3390/jcm13082273, 38673546 PMC11051044

[6] Giannopapas V Stefanou MI Zouvelou V Papagiannopoulou G Smyrni V Kosmidou M . Risk of diabetes mellitus in the myasthenia gravis: a systematic review and meta-analysis. J Clin Med. (2025) 14:4221. doi: 10.3390/jcm14124221, 40565966 PMC12194735

[7] Shao T Lu J Kang H Zhang Y Lan T Wang J. Diabetes mellitus in patients with myasthenia gravis: a systematic review and meta-analysis. Endocrine. (2025) 88:24–35. doi: 10.1007/s12020-024-04143-1 39729181

[8] Liu Y-D Tang F Li X-L Liu Y-F Zhang P Yang C-L . Type 2 diabetes mellitus as a possible risk factor for myasthenia gravis: a case-control study. Front Neurol. (2023) 14:1125842. doi: 10.3389/fneur.2023.1125842 37139075 PMC10149973

[9] Dong J Shi X Gu X-D Yang Y He Y-L Zhou Y . Diabetes exacerbates myasthenia gravis by enhancing pathological thymic output via the Gpr183-7alpha,25-Ohc axis. J Neuroinflammation. (2025) 22:250. doi: 10.1186/s12974-025-03571-8 41163004 PMC12570436

[10] Sharif S Sharif H Rehman J Fatima Z. Is a sedentary lifestyle a leading causal factor of obesity and distress in type 2 diabetes? A cross-sectional study in low-socioeconomic areas of Karachi, Pakistan. Bmj Public Health. (2023) 1:e000149. doi: 10.1136/bmjph-2023-000149, 40017838 PMC11816276

[11] Barker HL Morrison D Llano A Sainsbury CAR Jones GC. Practical guide to glucocorticoid induced hyperglycaemia and diabetes. Diab Ther. (2023) 14:937–45. doi: 10.1007/s13300-023-01393-6 PMC10037401 36961675

[12] Li T Yang CL Du T Zhang P Zhou Y Li X-L . Diabetes mellitus aggravates humoral immune response in myasthenia gravis by promoting differentiation and activation of circulating Tfh cells. Clin Immunol. (2022) 245:109141. doi: 10.1016/j.clim.2022.109141, 36270469

[13] Zhang P Yang CL Du T Liu Y-D Ge M-R Li H . Diabetes mellitus exacerbates experimental autoimmune myasthenia gravis via modulating both adaptive and innate immunity. J Neuroinflammation. (2021) 18:244. doi: 10.1186/s12974-021-02298-6, 34702288 PMC8549151

[14] Andersen LK Aadahl M Vissing J. Fatigue, physical activity and associated factors in 779 patients with myasthenia gravis. Neuromuscul Disord. (2021) 31:716–25. doi: 10.1016/j.nmd.2021.05.007, 34303571

[15] Datta N Hoke A. Repetitive Nerve Stimulation. Treasure Island (Fl): StatPearls (2025). 37603640

[16] Yang XG Guo HY Peng Z Luo HT Lu S. Application of electrophysiological techniques in assessing of neuromuscular junction-related disorders. World Neurosurg. (2024) 191:165–71. doi: 10.1016/j.wneu.2024.08.076, 39159673

[17] Kouyoumdjian JA Estephan EP. Electrophysiological evaluation of the neuromuscular junction: a brief review. Arq Neuropsiquiatr. (2023) 81:1040–52. doi: 10.1055/s-0043-1777749 38157872 PMC10756823

[18] Juel VC. Clinical neurophysiology of neuromuscular junction disease. Handb Clin Neurol. (2019) 161:291–303. doi: 10.1016/B978-0-444-64142-7.00055-2, 31307607

[19] Savelieff MG Elafros MA Viswanathan V Jensen TS Bennett DL Feldman EL. The global and regional burden of diabetic peripheral neuropathy. Nat Rev Neurol. (2025) 21:17–31. doi: 10.1038/s41582-024-01041-y 39639140 PMC13011988

[20] Ziegler D. Pathogenetic treatments for diabetic peripheral neuropathy. Diab Res Clin Pract. (2023) 206:110764. doi: 10.1016/j.diabres.2023.110764 38245327

[21] Narayanaswami P Sanders DB Wolfe G Benatar M Cea G Evoli A . International consensus guidance for management of myasthenia gravis: 2020 update. Neurology. (2021) 96:114–22. doi: 10.1212/WNL.0000000000011124 33144515 PMC7884987

[22] American Diabetes Association Professional Practice C. 2. Diagnosis and classification of diabetes: standards of care in diabetes-2024. Diab Care. (2024) 47:S20–42. doi: 10.2337/dc24-S002 PMC10725812 38078589

[23] Sanders DB Cao L Massey JM Juel VC Hobson-Webb L Guptill JT. Is the decremental pattern in Lambert-Eaton syndrome different from that in myasthenia gravis? Clin Neurophysiol. (2014) 125:1274–7. doi: 10.1016/j.clinph.2013.11.007, 24332471

[24] Huang C-R Chang W-N Chang H-W Tsai N-W Lu C-H. Effects of age, gender, height, and weight on late responses and nerve conduction study parameters. Acta Neurol Taiwanica. (2009) 18:242–9. 20329591

[25] Awang MS Abdullah JM Abdullah MR Tahir A Tharakan J Prasad A . Nerve conduction study of healthy Asian Malays: the influence of age on median, ulnar, and sural nerves. Med Sci Monit. (2007) 13:Cr330–2. 17599028

[26] Marfella R D'onofrio N Sardu C Scisciola L Maggi P Coppola N . Does poor glycaemic control affect the immunogenicity of the Covid-19 vaccination in patients with type 2 diabetes: the caveat study. Diabetes Obes Metab. (2022) 24:160–5. doi: 10.1111/dom.1454727 34494705 PMC8653151

[27] American Diabetes Association Professional Practice C. 2. Classification and diagnosis of diabetes: standards of medical care in diabetes-2022. Diab Care. (2022) 45:S17–38. 10.2337/dc22-S002 34964875

[28] Boldizsar F Berki T Miseta A Németh P. Effect of hyperglycemia on the basal cytosolic free calcium level, calcium signal and tyrosine-phosphorylation in human T-cells. Immunol Lett. (2002) 82:159–64. doi: 10.1016/S0165-2478(02)00032-9, 12008048

[29] Misra UK Kalita J Singh VK Kumar S. A study of comorbidities in myasthenia gravis. Acta Neurol Belg. (2020) 120:59–64. doi: 10.1007/s13760-019-01102-w 30972663 PMC7222966

[30] Chu HT Tseng CC Liang CS Yeh TC Hu LY Yang AC . Risk of depressive disorders following myasthenia gravis: a nationwide population-based retrospective cohort study. Front Psych. (2019) 10:481. doi: 10.3389/fpsyt.2019.00481, 31354544 PMC6629932

[31] Pegat A Gavoille A Bonjour M Cluse F Moussy M Svahn J . Is the decrement pattern in myasthenia gravis due to muscle-specific kinase antibodies different to that due to acetylcholine receptor antibodies? Neurophysiol Clin. (2025) 55:103092. doi: 10.1016/j.neucli.2025.103092, 40743586

[32] Tumurov DA Sanadze AG. Decrement pattern of M-response amplitude in the low-frequency repetitive nerve stimulation in the muscles of patients with myasthenia gravis and Lambert-Eaton myasthenic syndrome. Zh Nevrol Psikhiatr Im S S Korsakova. (2017) 117:93–6. doi: 10.17116/jnevro20171172193-96, 28374700

[33] Miralles F. Modelling the response to low-frequency repetitive nerve stimulation of myasthenia gravis and Lambert-Eaton myasthenic syndrome. Med Biol Eng Comput. (2016) 54:1761–78. doi: 10.1007/s11517-016-1462-4 27016366

[34] Baslo MB Deymeer F Serdaroglu P Parman Y Ozdemir C Cuttini M. Decrement pattern in Lambert-Eaton myasthenic syndrome is different from myasthenia gravis. Neuromuscul Disord. (2006) 16:454–8. doi: 10.1016/j.nmd.2006.05.009, 16806929

[35] Zhang D Zhao Y Yan C Cao L Li W. Cmap decrement by low-frequency repetitive nerve stimulation in different hand muscles of Als patients. Neurol Sci. (2019) 40:2609–15. doi: 10.1007/s10072-019-04027-7, 31375938

[36] Fu LL Yin HX Liu MS Cui L-Y. Study on variation trend of repetitive nerve stimulation waveform in amyotrophic lateral sclerosis. Chin Med J. (2019) 132:542–50. doi: 10.1097/cm9.0000000000000117, 30807353 PMC6415996

[37] Luigetti M Modoni A Lo Monaco M. Low rate repetitive nerve stimulation in Lambert-Eaton myasthenic syndrome: peculiar characteristics of decremental pattern from a single-Centre experience. Clin Neurophysiol. (2013) 124:825–6. doi: 10.1016/j.clinph.2012.08.026, 23036181

[38] Sloan G Selvarajah D Tesfaye S. Pathogenesis, diagnosis and clinical management of diabetic sensorimotor peripheral neuropathy. Nat Rev Endocrinol. (2021) 17:400–20. doi: 10.1038/s41574-021-00496-z, 34050323

[39] Selvarajah D Kar D Khunti K Davies MJ Scott AR Walker J . Diabetic peripheral neuropathy: advances in diagnosis and strategies for screening and early intervention. Lancet Diabetes Endocrinol. (2019) 7:938–48. doi: 10.1016/s2213-8587(19)30081-6, 31624024

[40] Andersen H. Motor dysfunction in diabetes. Diabetes Metab Res Rev. (2012) 28:89–92. doi: 10.1002/dmrr.2257 22271730

[41] Estrada-Bonilla YC Castro P Luna GLF Castro PATS Souza ABA Santos GS . Reaching task performance is associated to neuromuscular junction adaptations in rats with induced diabetes mellitus. Braz J Med Biol Res. (2020) 53:e8763. doi: 10.1590/1414-431X20208763, 32520205 PMC7279698

[42] Muramatsu K Niwa M Nagai M Kamimura T Sasaki S Ishiguro T. The size of motoneurons of the gastrocnemius muscle in rats with diabetes. Neurosci Lett. (2012) 531:109–13. doi: 10.1016/j.neulet.2012.10.031, 23127853

[43] Morrow TJ. Animal models of painful diabetic neuropathy: the Stz rat model. Curr Protoc Neurosci. (2004):18. doi: 10.1002/0471142301.ns0918s29 18428614

[44] Fahim MA Hasan MY Alshuaib WB. Early morphological remodeling of neuromuscular junction in a murine model of diabetes. J Appl Physiol. (2000) 89:2235–40. doi: 10.1152/jappl.2000.89.6.2235, 11090573

[45] Fahim MA El-Sabban F Davidson N. Muscle contractility decrement and correlated morphology during the pathogenesis of streptozotocin-diabetic mice. Anat Rec. (1998) 251:240–4. doi: 10.1002/(SICI)1097-0185(199806)251:2<240::AID-AR13>3.0.CO;2-O 9624455

